# Variants of SARS-CoV-2: Influences on the Vaccines’ Effectiveness and Possible Strategies to Overcome Their Consequences

**DOI:** 10.3390/medicina59030507

**Published:** 2023-03-05

**Authors:** Ali A. Rabaan, Shamsah H. Al-Ahmed, Hawra Albayat, Sara Alwarthan, Mashael Alhajri, Mustafa A. Najim, Bashayer M. AlShehail, Wasl Al-Adsani, Ali Alghadeer, Wesam A. Abduljabbar, Nouf Alotaibi, Jameela Alsalman, Ali H. Gorab, Reem S. Almaghrabi, Ali A. Zaidan, Sahar Aldossary, Mohammed Alissa, Lamees M. Alburaiky, Fatimah Mustafa Alsalim, Nanamika Thakur, Geetika Verma, Manish Dhawan

**Affiliations:** 1 Molecular Diagnostic Laboratory, Johns Hopkins Aramco Healthcare, Dhahran 31311, Saudi Arabia; 2 College of Medicine, Alfaisal University, Riyadh 11533, Saudi Arabia; 3 Department of Public Health and Nutrition, The University of Haripur, Haripur 22610, Pakistan; 4 Specialty Paediatric Medicine, Qatif Central Hospital, Qatif 32654, Saudi Arabia; 5 Infectious Disease Department, King Saud Medical City, Riyadh 7790, Saudi Arabia; 6 Department of Internal Medicine, College of Medicine, Imam Abdulrahman Bin Faisal University, Dammam 34212, Saudi Arabia; 7 Department of Medical Laboratories Technology, College of Applied Medical Sciences, Taibah University, Madinah 41411, Saudi Arabia; 8 Pharmacy Practice Department, College of Clinical Pharmacy, Imam Abdulrahman Bin Faisal University, Dammam 31441, Saudi Arabia; 9 Department of Medicine, Infectious Diseases Hospital, Kuwait City 63537, Kuwait; 10 Department of Infectious Diseases, Hampton Veterans Administration Medical Center, Hampton, VA 23667, USA; 11 Department of Anesthesia, Dammam Medical Complex, Dammam 32245, Saudi Arabia; 12 Department of Medical Laboratory Sciences, Fakeeh College for Medical Science, Jeddah 21134, Saudi Arabia; 13 Clinical Pharmacy Department, College of Pharmacy, Umm Al-Qura University, Makkah 21955, Saudi Arabia; 14 Infection Disease Unit, Department of Internal Medicine, Salmaniya Medical Complex, Ministry of Health, Kingdom of Bahrain, Manama 435, Bahrain; 15 Al Kuzama Primary Health Care Center, Al Khobar Health Network, Eastern Health Cluster, Al Khobar 34446, Saudi Arabia; 16 Organ Transplant Center of Excellence, King Faisal Specialist Hospital and Research Center, Riyadh 11211, Saudi Arabia; 17 Gastroenterology Department, King Fahad Armed Forces Hospital, Jeddah 23831, Saudi Arabia; 18 Pediatric Infectious Diseases, Women and Children’s Health Institute, Johns Hopkins Aramco Healthcare, Dhahran 31311, Saudi Arabia; 19 Department of Medical Laboratory Sciences, College of Applied Medical Sciences, Prince Sattam bin Abdulaziz University, Al-Kharj 11942, Saudi Arabia; 20 Pediatric Department, Safwa General Hospital, Eastern Health Cluster, Safwa 31921, Saudi Arabia; 21 Department of Family Medicine, Primary Health Care, Qatif Health Cluster, Qatif 32434, Saudi Arabia; 22 University Institute of Biotechnology, Department of Biotechnology, Chandigarh University, Mohali 140413, India; 23 Department of Experimental Medicine and Biotechnology, Post Graduate Institute of Medical Education and Research (PGIMER), Chandigarh 160012, India; 24 Department of Microbiology, Punjab Agricultural University, Ludhiana 141004, India; 25 Trafford College, Altrincham, Manchester WA14 5PQ, UK

**Keywords:** SARS-CoV-2, COVID-19, breakthrough infections, neutralizing antibodies (NAbs), Omicron, vaccines, variants

## Abstract

The immune response elicited by the current COVID-19 vaccinations declines with time, especially among the immunocompromised population. Furthermore, the emergence of novel SARS-CoV-2 variants, particularly the Omicron variant, has raised serious concerns about the efficacy of currently available vaccines in protecting the most vulnerable people. Several studies have reported that vaccinated people get breakthrough infections amid COVID-19 cases. So far, five variants of concern (VOCs) have been reported, resulting in successive waves of infection. These variants have shown a variable amount of resistance towards the neutralising antibodies (nAbs) elicited either through natural infection or the vaccination. The spike (S) protein, membrane (M) protein, and envelope (E) protein on the viral surface envelope and the N-nucleocapsid protein in the core of the ribonucleoprotein are the major structural vaccine target proteins against COVID-19. Among these targets, S Protein has been extensively exploited to generate effective vaccines against COVID-19. Hence, amid the emergence of novel variants of SARS-CoV-2, we have discussed their impact on currently available vaccines. We have also discussed the potential roles of S Protein in the development of novel vaccination approaches to contain the negative consequences of the variants’ emergence and acquisition of mutations in the S Protein of SARS-CoV-2. Moreover, the implications of SARS-CoV-2’s structural proteins were also discussed in terms of their variable potential to elicit an effective amount of immune response.

## 1. Introduction

Safe and effective vaccination has been critical in the ongoing battle against Severe Acute Respiratory Syndrome Coronavirus 2 (SARS-CoV-2). The development of precise vaccine platforms in such a short period is a testament to global scientific prowess, and, as of 9 June, 2021, more than 2,156,550,767 doses of the COVID-19 vaccine have been given across the five continents [1]. Unfortunately, reports on variants of SARS-CoV-2 brought about by mutations with enhanced virulence, pathogenicity, and the ability to detrimentally affect host immune systems, especially the antibodies produced after COVID-19 vaccination, is a matter of concern and scientific deliberation.

However, the available published data divulge that the current vaccines could still be effective in preventing severe infection and death in people infected with the recent variants of SARS-CoV-2, such as Omicron and Delta [2,3,4,5,6]. Multiple studies have shown several advantages of the numerous mutations of the Omicron variant of the SARS-CoV-2 virus [7,8,9]. The Omicron variant and its subvariants evolved by evolutionary processes that may lead to a number of significant modifications in the virus’s characteristics, such as immunological escape from the nAbs produced by the administration of the vaccines [10,11,12]. The high frequency of mutations has also been linked to improved proteolytic priming with transmembrane serine protease 2 (TMPRSS2) and the increased binding capacities of S Protein to the angiotensin converting enzyme 2 (ACE2) receptor [9,10,13,14,15]. The higher number of mutations in the Omicron variant have also been associated with improved resistance to endosomal restriction factors, specifically IFITM proteins, which enables the variant’s more effective cellular invasion via the endocytic route [16]. Additionally, the modifications may make it more likely for spike protomers to adopt an up configuration to interact with ACE2, and may increase the stability of a down configuration to prevent contact with nAbs [12,17,18].

A variant can be defined as an isolate whose genome sequence differs from that of the reference virus. Thus, the variants share an identical inherited set of distinct mutations and are classified based on a lineage, i.e., the type of mutations that resulted in the origination of a new lineage of SARS-CoV-2. From this perspective, it is crucial to understand the mutational dynamics of SARS-CoV-2 and its effects on the vaccines that are currently available [19]. Studies have deciphered that a typical SARS-CoV-2 virus accrues, on average, one or two single-nucleotide genomic mutations in a period of 30 days [3,4]. This is just 50% of the rate of the mutational dynamics of influenza and 25% of the AIDS human immunodeficiency virus (HIV). The retarded mutational dynamics of SARS-CoV-2 could perhaps be credited to the specific exoribonuclease (ExoN) present in the genome of coronaviruses (CoVs), since inactivation of this ExoN has demonstrated a twenty-fold increase in the mutation rates [4,5].

### Effects of Mutations on Variants’ Characteristics

Some significant mutations documented in SARS-CoV-2 include the ‘N501Y’, in which the spike protein (S Protein) 501st amino acid is swapped from N (asparagine) to Y (tyrosine) and assists the virus to attach more rigidly to human cells. Substitution of histidine at 681 positions instead of proline results in a change in an amino acid on the stem region of the spike of SARS-CoV-2 that triggers infected host cells to give rise to new spike proteins. ‘H69-V70’ is yet another mutation caused by the deletion in the 69th and 70th position of the a/a in spike protein, and it changes the shape of the spike, facilitating the virus to escape from some antibodies [4,20,21].

The ‘Y144/145’ mutation caused by the elimination of the 144th or 145th amino acids (tyrosine, Y) in the S Protein area challenges the effectual attachment of antibodies with the SARS-CoV-2 virus. ‘ORF8 Q27stop’ is another important mutation that involves the ORF8, a 121 amino acid protein whose function is yet to be completely deciphered. The ‘D614 G’ mutation has a moderate documented impact on transmissibility brought about by the alteration in the spike protein, where G (glycine) is substituted by D (aspartic acid). ‘E484K’, often referred to as the “escape mutation,” brought about by a swap wherein the glutamic acid (E) is substituted by lysine (K) at position 484, shields the virus from at least one type of monoclonal antibody. ‘L452R’, initially detected in Denmark, is yet another mutation that has been detected in various lineages [4,22,23]. Moreover, the presence of mutations such as H69/V70 deletions, the substitution of lysine instead of threonine (T478K), and the insertion of alanine at 484 positions instead of glutamic acid (E484A) in already reported variants of concern (VOC-) have been associated to the variants’ enhanced capacity to evade the defense mechanism of the body [21]. The higher ACE2 receptor binding capacity of the S Protein has been linked to the N501Y mutation. Furthermore, the S Protein’s capacity to attach to the ACE2 receptor was markedly enhanced by the Q498R mutation with N501Y. These modifications make it simple for the Omicron variant to penetrate the host cell [10].

Important mutations in the Omicron variant [7] include A76V, Y145del, G339D, N440K, G446S, E484A, Q493R, G496S, Q498R, Y505H, T547K, H655Y, N679K, N764K, D796Y, N856K, Q954H, N969K, and L981F [7]. It is interesting to note that similar changes have been observed in other types, albeit with varying effects. Moreover, the Omicron variant acquired new mutations that increased its ability to spread [24,25]. Key amino acid alterations in the RBD of the S Protein [7] include G339D, S371L, S373P, S375F, K417N, N440K, G446S, S477N, T478K, E484A, Q493R, G496S, Q498R, N501Y, and Y505H [7,24,25]. Such alterations may also be linked to elevated affinities of the S Protein for the ACE2 receptor [26]. The crucial step in obtaining access into the host cell is the binding of the ACE2 receptor to the S Protein of the SARS-CoV-2 [27]. Human transmembrane protease serine 2 (TMPRSS2) cleaves the S Protein after it interacts with ACE2 receptors on the cell membrane. The S Protein is split up into its S1 and S2 subunits by TMPRSS2, which in turn makes the RBD on the S1 subunit available for interactions [28,29,30]. Consecutively, the S2 domain undergoes structural modifications that assist in the union of viral and cellular membranes [31,32]. It is noteworthy that according to studies using electron microscopy, the SARS-CoV-2 S Protein has a binding affinity to ACE2 that is around 10–20 times larger than that of the S proteins from other SARS-CoVs [30,33].

The S Protein of SARS-CoV-2 must be split at the S1-S2 and S2 locations in order to enter host cells [7]. Furin24, type II transmembrane serine protease (TMPRSS2), or cathepsin L are the enzymes responsible for this cleavage [34,35]. The TMPRSS2 and cathepsin L breakdown at the S2 site facilitate two distinct SARS-CoV-2 entry pathways [7]. However, mutations in certain variants are considered plausible reasons behind the changes in such entry pathways [7]. Due to it being expressed on the cell membrane, TMPRSS2 promotes the invasion via the plasma membrane as opposed to cathepsin L in the endosome, which favors the endosomal pathway [35,36]. Six different mutations in the subunit 2 (S2) of the S Protein of the Omicron variant, notably N764K, D796Y, N856K, Q954H, N969K, and L981F, were linked to variations in viral entrance into the cellular machinery and modes of transmission [37,38,39]. Recent investigations have shown that the Omicron variant favors the endosomal entry pathway over the plasma membrane entrance route [40]. Infection by the Omicron spike pseudotyped virus was likewise shown to be restricted in cells that express the transcription factor TMPRSS2 but increased in cells that support an endosomal route for entry [7,40].

Recent findings suggest that genetic alterations to the Omicron S protein non-RBD may alter the mode of viral entry into host cells, which is associated with a shift in cellular tropism away from TMPRSS2-expressing cells. These findings also demonstrate why, in contrast to other VOCs, such as the Alpha, Beta, and Delta variants, Omicron replicates more rapidly in the upper respiratory system than in the lower respiratory tract [40,41,42,43]. It seems that the Omicron variety also has three significant mutations, including P681H, H655Y, and N679K, in the furin cleavage region. It is known that changes such as P681H at the polybasic cleavage site (PBCS), which are also present in other VOCs such as Alpha and Gamma, make it easier for the S protein to be digested by furin and may thus make the organism more pathogenic [44]. Hence, altogether, this information suggests that the mutations or alterations in the viral genome led to drastic changes in the characteristics of their nature to infect and disseminate among populations. As per the mutations and their impacts on the pathogenicity and transmission, many government bodies have classified the variants of SARS-CoV-2 into various categories. The following section will highlight the same.

## 2. Classification of Variants of SARS-CoV-2

SARS-CoV-2 variants could be classified into four different groups, i.e., variants of interest (VOIs), variants of high consequence (VOHCs), variants under monitoring (VUMs), and variants of concern (VOCs) by the US Department of Health and Human Services [17,18,19], and all five VOCs have been further categorized as α, β, γ, δ, and Omicron variants by the World Health Organization (WHO). The Omicron variant has quickly competed with other VOCs and spread across the world [10] [Table 1].

### 2.1. Variants of Interest (VOIs)

VOI is a variant that has genetic markers specifically linked with changes to host receptor binding, exhibiting reduced antibody neutralization production versus a previous infection by the reference virus or vaccination, and showing a reduced response to hitherto effective treatments, causing a potential diagnostic impediment, and carrying on it a label of predictive upsurge in infection. These include the B.1.525 lineage brought about by the spike protein (S protein) substitutions 69del, 144del, 70del, A67V, D614G, E484K, F888L, andQ677H, which was first detected in the United Kingdom and Nigeria in December 2020; the B.1.526 lineage brought about by spike protein substitutions A701V, D253G, D614G, E484K, L5F, T95I, and S477N, which was first detected in the United States in November 2020; the B.1.526.1 lineage brought about by spike protein (S protein) substitutions D80G, D614G, D950H, 144del, F157S, L452R, T791I, and T859N, which was first detected in the United States in October 2020; the B.1.617 lineage, brought about by spike protein (S protein) substitutions D614G, L452R, and E484Q, which was first noticed in India in February 2021; the B.1.617.1 lineage, brought about by spike protein (S protein) substitutions, i.e., D614G, E484Q, E154K, G142D, L452R, P681R, Q1071H, and T95I, which was first identified in India in December 2020; B.1.617.3 lineage, brought about by spike protein (S protein) substitutions D614G, D950N, E484Q, G142D, L452R, P681R, and T19R, which was first spotted in India in October 2020; and the P.2 lineage, brought about by spike protein (S protein) substitutions D614G, E484K, F565L, andV1176F, which was first identified in Brazil in April 2020 [45,46,47,48,49,50,51].

### 2.2. Variants of Concern (VOCs)

A variant for concern (VOC) is a variant that has strong evidence of an intensified transmissibility; severity of the disease symptoms, including a higher number of hospitalizations and deaths; shows a significant decrease in neutralization by post-vaccination and convalescent sera; displays the significant reduction in the efficacy of existing treatments and vaccines; and poses notable diagnostic challenges, which lead to insufficiency in the diagnosis of the variant. Up to this point, the WHO has identified five VOC variants, including α, β, γ, δ, and Omicron. The Omicron variant has quickly spread around the globe and fought against all the VOCs. According to the most recent information [10], the Omicron variant (B.1.1.529) contains >30 mutations in the S Protein compared to other VOCs such as α (B.1.1.7), β (B.1.351) and δ (B.1.617.2). Significant changes to the N-terminal domain (NTD) and receptor-binding domain (RBD) of the S Protein have been linked to greater resistance to nAbs and transmission [10]. Interestingly, these VOCs could necessitate serious emergency public health engagements, including immediate notification of the detected variant to the World Health Organisation (WHO) under the regulation of International Health to CDC, and the regional and governmental authorities to control and end the spread.

These variants could also compel improved testing and investigation of the efficacy of pre-existing vaccines and treatments as well as force the deployment of newer diagnostics and the modification or production of suitable vaccines/therapeutics. These include the B.1.1.7 lineage, brought about by S Protein substitutions deletion at 69, 70, 144, N501Y, E484K, A570D, P681H, S982A, K1191N, S494P, D1118H, D614G, and T716I, which was first detected in the United Kingdom [52,53]; the B.1.351 lineage, brought about by spike protein substitutions D2, 241del, 243del, D614G, D80A, E484K, 15G, 242del, K417N, N501Y, and A701V, which was first found in South Africa [52,53,54]; the B.1.427 lineage, brought about by spike protein substitutions L452R and D614G, and observed for the first time in California, USA; the B.1.429 lineage, first observed in California, USA, due to substitutions such as L452R, S13I, W152C, and D614G in the spike protein [54,55,56]; the B.1.617.2 lineage (Delta), brought about by spike protein substitutions T19R, P681R, G142D, D614G, R158G, L452R, T478K, 156del, 157del, and D950N, which was first detected in India in December 2020 [41]; and the P.1 lineage, due to substitution in spike protein (L18F, D138Y, T20N, E484K, D614G, P26S, R190S, T1027I, K417T, N501Y, H655Y), which was first detected in Japan and Brazil [57,58,59,60,61,62,63,64,65,66,67].

### 2.3. Variants of High Consequence (VOHCs)

A variant of high consequence is explained as a variant for which there is absolute evidence that its prevalence has significantly decreased the effectiveness of medical countermeasures (MCMs) and preventive measures compared to the previously circulating variants. A variant of high consequence can also cause the established failure of diagnostic protocols and severe reduction in efficiency of the currently available vaccines and jeopardize the (EUA) Emergency Use Authorization and approved therapeutics, perhaps with harsher clinical manifestations and a higher number of hospitalizations. To this date, none of the variants of high consequence have been recorded [68].

## 3. Influence of Variants’ Emergence on Vaccine Effectiveness

VOCs, especially the Delta variant, may affect the neutralising activity of vaccine-elicited Abs and MAbs, which might result in a mild-to-significant decrease in efficiency for COVID-19 vaccines and immunotherapeutic treatment [69,70]. The existing vaccination strategies failed to prevent the outbreak of Omicron variants [59,62,66,71,72,73]. NAbs in sera from those who received a 2-dose Ad26.COV2.S (Johnson & Johnson) vaccine were considerably less efficient against the Omicron variant than the primary strain of SARS-CoV-2. A luciferase-based pseudo virus neutralisation experiment revealed a dramatic decline in the antibody-mediated immune response, 20 × 102, when compared to the original strain, which was 184 × 103 on the eighth day following vaccination [46]. Similar researchers, however, have shown that cellular immunity produced by existing vaccines against SARS-CoV-2 is largely conserved to the SARS-CoV-2 Omicron spike protein [46]. Vaccination with Ad26.COV2.S or BNT162b2 resulted in substantial spike-specific CD8+ and CD4+ T cell responses as well as significant cross-reactivity against both the Delta and Omicron variants in both the central and effector memory cell subpopulations [46].The serum neutralizing ability of individuals receiving BNT162b2 (Pfizer/BioNTech) was diminished 35-fold against BA.1 compared to D614G variant [62,66,74]. Additionally, it was not effective against BA.2 and BA.3 [75]. However, the booster doses of vaccines proved beneficial in increasing the efficacy of serum-neutralizing titers against Omicron [62,71,76].

A Phase III trial of Covaxin (BBV152), an inactivated SARS-CoV-2 vaccine, established by Bharat Biotech, India, confirmed its potential effectiveness against symptomatic cases (77.8%) and the Delta variant (68.2%) [77]. However, the convalescent serum of recipients of BBV152 was not able to neutralise the P.1 lineage [78].

Studies have shown that ChAdOx1 nCoV-19 (AZD1222) is effective against Alpha (74.5%), Delta (67%) [79], and Gamma (77.9%) [80]; however, not against Beta (10.4%) [5]. Further, this vaccine was associated with some cases of thrombosis and thrombocytopenia syndrome (TTS), blood clot events, and deaths, causing the suspension of the use of this vaccine in many European and Asian countries [81].

A renowned mRNA-based vaccine BNT162b2 was created by Pfizer and is often utilized in the immunization programs of nations. Two booster doses of this vaccine give a similar level of protection against Delta, but recent comparative studies have cast doubt on the vaccine’s effectiveness [76,82]. The efficacy of the BNT162b2 and ChAdOx1 nCoV-9 vaccinations was shown to be lower in those who had the Delta variation of the virus than in those who had the other VOCs. It is critical to keep in mind that these outcomes were attained in patients who received only one dose of the vaccine [70].

Further investigation with two doses of the vaccination has demonstrated the apparent efficiency of the primary vaccines against the δ variant. Two doses of the BNT162b2 vaccination were effective in persons with the Alpha version, and 88% in those with the Delta version. Two doses of the ChAdOx1 nCoV-19 vaccine were shown to be 74.5% effective in those with the Alpha form and 67.0% effective among individuals with the δ variant. Upon receiving the two vaccine doses, minor changes in vaccine efficacy were observed between the δ and α variants. Absolute disparities in vaccination effectiveness become more evident after the first dose. This outcome will aid the efforts to increase vaccination uptake among a vulnerable subset of individuals through the administration of two doses [79]. According to several investigations, three doses of BNT162b2 mRNA seem to be necessary to protect against Omicron-driven COVID-19 [83,84,85]. Surprisingly, Gao et al. proposed that pre-existing SARS-CoV-2 spike-specific CD8+ and CD4+ T cell responses are usually intact against Omicron, especially after BNT162b2 vaccination [86].

Additional research revealed that two doses of the BNT162b2 or ChAdOx1 nCoV-19 vaccination only partially protected against the omicron variant. Upon receiving a BNT162b2 or mRNA-1273 vaccine booster shots, the protection from the BNT162b2 or ChAdOx1 nCoV-19 primary vaccination increased but ultimately wore out [87].

However, the serum from individuals administered triple doses of ChAdOx1 (Oxford/AstraZeneca) or BNT162b2 showed a decreased efficacy against BA.4/5, contrary to BA.1 and BA.2 [88]. Recent research by Zou et al. 2022 found that following the complete dosage of the SARS-CoV-2 vaccine, the immune protection diminishes with time against the Omicron variant.

The Omicron form could not be neutralised by more than half of the mRNA-1273 recipients’ plasma, leading to GMTs that were 43 times lower [60]. According to Pajon et al., neutralisation titers against the Omicron version of the mRNA-1273 vaccination were 35 times lower than those against the D614G variant after the first two doses of the vaccine. On the other hand, neutralisation titers against the Omicron variant were 20 times greater following the booster dose of the mRNA-1273 immunisation than following the second dosage, indicating that the risk of relapse is significantly reduced. Six months following the booster injection, neutralisation titers against the Omicron variant decreased [61].

The effectiveness of the serum from people who received an mRNA vaccination against Omicron was evaluated by Edara et al.: using a live viral experiment, they noticed a 30-fold decrease in neutralising activity against the Omicron 2–4 weeks after receiving a primary batch of immunisations, but six months after the first two vaccination doses, no neutralising activity against the Omicron was found, and, in addition, they found that following a booster injection (third dosage), naive individuals’ neutralising activity against Omicron decreased fourteen-fold [62]. This implies that the vaccination’s effectiveness has been compromised by the appearance of variations, which calls for the administration of booster doses of the vaccine at progressively longer intervals.

## 4. The SARS-CoV-2 Structural Proteins and their Inference in the Vaccine’s Development

**Figure 1:**
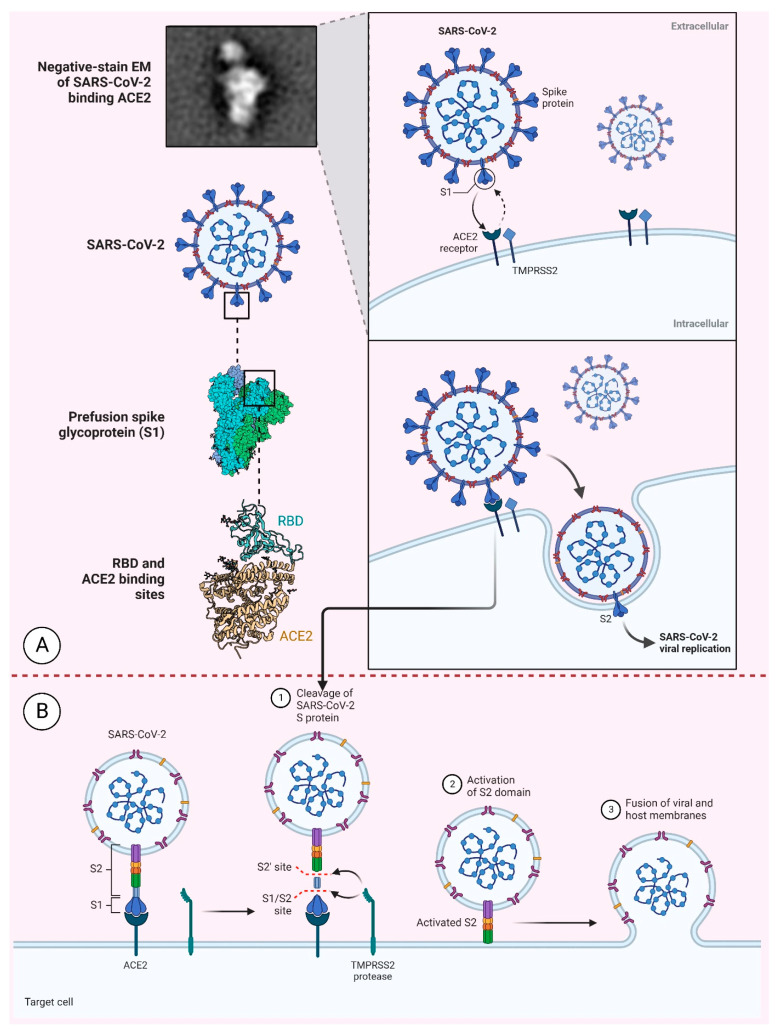
The schematic representation of the SARS-CoV-2 entry into the host cell. (**A**) Representation of the S Protein and its various regions such as receptor binding domain (RBD); (**B**) Binding of the S Protein with the ACE2 receptor of the host cell. After binding of the S Protein with the ACE2 receptor, there is cleavage of the S Protein into S1 and S2 sites. Activated S2 domain helps in the fusion of the viral particle with the cell membrane. [The figure was created with the templates available in BioRender.com, accessed on 25 January 2023].

S Protein, M-protein, E-protein, and N-nucleocapsid protein (ribonucleoprotein core) are the main structural vaccine target proteins for COVID-19 [63,64]. Sixteen non-structural proteins (nsp1–16) and nine accessory proteins encoded by the virus are additional targets [65]. S Proteins help viruses perceive host cellular receptors and enter [Figure 1]. Hence, the S Protein is the main target of SARS-CoV-2 vaccines, and it exists as a homotrimer in the viral envelope with its membrane-distal S1 and S2 subunits (which are membrane-proximal). S1′s receptor-binding domain modulates receptor recognition [66,67,89]. It is imperative to mention here that the N-terminal domain (NTD) of the subunit S1 could also serve as a receptor-interacting domain, as in the case of the mouse hepatitis virus [90,91,92]. The S2 subunit facilitates membrane fusion during host entry and comprises the fusion peptide, connecting region HR1 and HR2 (heptad repeats) as a ‘helix-turn-helix’ construct around a central helix [93].

Recent molecular evidence of this structure has advocated a paradigm where there occurs a rearrangement of this S Protein subsequent to the identification of the host cell receptor [94,95]. Recent research has shown that upon the RBD’s interaction with ACE2, the S1 subunit dissociates while the S2 subunit simultaneously refolds, allowing the FP to protrude for effective membrane fusion. The S2 subunit then folds and enters a long helical bundle post-fusion conformation after the FP is inserted into the host cell membrane [60].

More recently, research has focused on strategies that aim to keep the spike-protein in its pre-fusion form, especially in the wake of studies on two proline substitutions at the top of the central helix, HR1 of MERS-CoV and SARS-CoV, and HKU1 that can keep the S Proteins in the pre-fusion conformation [96]. Studies have further shown that this hybrid antigen ‘S-2P’ could produce higher NAb titres than the S protein. Thus, the S-2P hybrid strategy is a potential vaccine target against SARS-CoV-2. The S-2P has further facilitated the generation of a new S protein ectodomain, ‘HexaPro’, consisting of six proline substitutions, with the inclusion of the two from S-2P positioned at the N termini of helices or flexible loops in the CR, FP, and HR1 [97]. Such positioning has been shown to promote limiting the reorganization of the S2 subunit structure, stabilizing the pre-fusion spike protein and producing a tenfold increase in the titre expression than S-2P [97]. Hence, stratagems to fix the S-protein to the pre-fusion conformation are a promising avenue.

Interestingly, NAbs have been found capable of targeting the S Protein at various stages of the viral entry, and RBD has been the major target of these Nabs, thwarting the viral receptor binding in the host [31,98,99], whilst almost all NAbs from the vaccines versus the SARS-CoV-2 have RBD as their target [32,100,101,102,103,104,105,106,107,108,109,110,111]. Many of the NAbs for SARS-CoV-2 have been observed to append to the RBD, thereby blocking the interaction of the RBD with hACE2 and thwarting the virus-host attachment during an infection episode [112]. Thus, the RBD is an alluring vaccine target with a guaranteed evocation of robust antibodies without any hazards of antibody-dependent enhancement of infection typically arbitrated by faint NAbs [113,114,115,116,117]. Further, RBD has also been shown to have T-cell response epitopes [118,119,120,121,122,123]. However, the petite molecular dimension and the probable existence of the same as multiple complexes, such as monomers or dimers, impose definite limits for the utilization of RBD as an effectual target. Various manoeuvres are being attempted now to trounce these disadvantages, such as magnifying the size of the antigen by fusion of the RBD with a fragment crystallizable region (Fc) [124,125,126,127,128] and multiplication of copies of the RBD (multimerization) [129,130,131]. A more recent study has suggested a dimeric design of the beta-CoV antigen RBDs that can be employed against SARS-CoV-2, since the RBDs have been shown to form dimers in solution naturally [132,133,134,135,136]. Studies have also demonstrated the possibility of homogeneous RBD-dimers as single chain repeats that would induce a ten-to-hundred-fold amplification of NAbs titres than the RBD-monomer [132,137].

Recent studies have demonstrated that the NAbs could also target the NTD, as stated previously [137], and the S1 protein-NTD has been shown to have Coronavirus NAbs epitopes [138,139]. Studies have shown that though the NAbs that target the NTD could not block the binding of the receptor directly, they could hinder the binding of the receptor binding [138,139] and curtail the previously discussed conformational modifications that occur during the process of pre-fusion to post-fusion alteration in the S-protein during infection. Though the NAbs that target NTD have been established to only display diminutive neutralizing efficacy compared to RBD-NAbs [49], NTD protein has been revealed to elicit definitive NTD-NAbs and T-cell responses with a decrease in lung-related abnormalities during infection [140].

The peptides of major interest in the S2 subunit capable of thwarting a viral fusion with target cells during infection are those elicited by HR1 or HR2 of the S2 subunit. Studies have established the effectual neutralization of the S2 of SARS-CoV-2 with NAbs [141,142,143], despite the expansive N-glycan shielding of the subunit S2 that renders it a difficult target for immune detection with lower NAb titers compared to the S1 and the RBD [144,145,146]. It is true that the subunit S2 as a sole entity might not be an operative humoral response target, but relative sequence conservation of the subunit S2 amongst various species of the virus makes it a candidate for recognition by CD4+ T cells and cross-reactive antibodies that can detect SARS-CoV-2 and various other human coronaviruses [147,148].

There has been little exploratory focus on the M, E, or N proteins in contrast to S. The M and E proteins have seldom displayed strong immunogenic elicitation of humoral responses, perhaps due to their petite ectodomains and molecular dimensions that are insufficient for immune recognition [149]. Yet, the relative sequence conservation of the M and E proteins amongst various species of the virus, i.e., SARS-CoV, SARS-CoV-2, and MERS-CoV, makes it a candidate for recognition by CD4+ T cells and cross-reactive antibodies [150]. The N protein, on the other hand, has been shown to be amply immunogenic during previous Corona episodes with established T cell epitopes [151]. Even though they failed against the SARS-CoV-2, N-target antibodies have been indicated to be effective in the mouse Hepatitis virus, which is another Coronavirus [152,153]. N protein has been demonstrated to elicit good T-cell immune responses (CD4+ and CD8+) [154]. T cell (CD8+) epitopes-specific to N protein in chicken IBV infection [155], and the Venezuelan Equine Encephalitis Virus replicon particles with a CD4+ T cell epitope specific to N have been shown to be fully effectual and immunologically protective for SARS-CoV infection [156]. Due to the conserved protein sequences between viruses, these virus replicon particles also provide some cross-protection against MERS-CoV, resulting in a decreased viral load [156]. Since previous vaccine studies on SARS-CoV expressing the N protein have reported infection-induced pneumonia due to enhanced Pulmonary Eosinophil Infiltration and Th-2 cell responses [157,158], causing a risk of Enhanced Respiratory Syncytial Virus Disease (ERD), vaccines based on the N protein have received little or no attention against SARS-CoV-2 [137,159].

## 5. Recent Strategies in the Vaccine Developments

**Figure 2:**
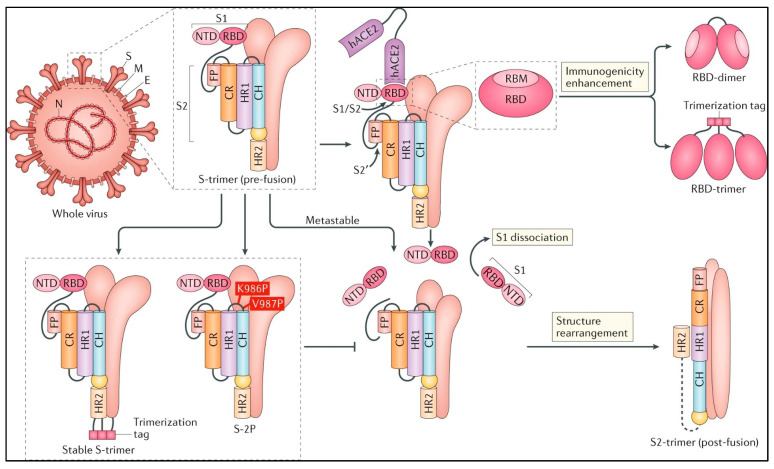
The principal targets of candidates for the COVID-19 vaccination; Source: [137].

A ‘Pan-corona Vaccine’, or at least a Pan-SARS-CoV-2 vaccine that will serve as a booster vaccine, seems to be the global stratagem now. In January 2021, Moderna began researching the SARS-CoV-2 B.1.351 variant, first discovered in South Africa [140,141]. Three vaccine candidates expressing the S Protein are entering Phase III clinical studies. The vaccine proposal from China is based on human adenovirus type 5 (Adn5). In contrast, the vaccine candidates from the United Kingdom are based on a recombinant chimpanzee adenovirus (AdnV), ChAdOx1, and the vaccine approach from Russia is based on recombinant human Ad26 and Ad5 [146]. RBD-based vaccines employ a protein sub-unit method [117], whereas the vaccine ARCoV encodes the RBD of the SARS-CoV-2 delivered via lipid nanoparticles (NPS) [147] [Figure 2].

Inactivated vaccines have already gained a lot of interest due to their ability to trigger immune responses comparable to those seen when viruses are exposed [160]. Given the absence of active genetic material, these vaccines include complete virus particles with no replication capabilities. Such vaccines are made utilizing viral inactivation techniques, including chemical agents such as formaldehyde, phenol, and glutaraldehyde; radiations such as UV, Xray, and Gamma; and physical means such as heat, pressure, and pH [160,161].

Nucleic acid-based vaccines, which include RNA vaccines, are novel forms of vaccinations [162]. These vaccinations include an mRNA strand that codes for a particular antigen/protein. When transported into living organisms, they may be translated into viral proteins. The viral protein might be displayed on the cell surface, where it is detected by immune system elements to elicit an immunological response [162]. RNA-based vaccines for viral illnesses such as influenza and rabies have already been investigated [161,162]. There are primarily two categories of viral vector-based vaccinations. Non-replicating vector vaccines do not manufacture any new viral particles; they only produce the vaccine protein. At the same time, replicating vector vaccines could create new virus particles and also infect cells. The SARS-CoV-2 vaccines currently under research employ non-replicating viral vectors [52,53].

Furthermore, Virus-like particles (VLPs) have gained much attention recently as they elicit a substantial amount of immune response as compared to conventional vaccines. In order to explain their strong immunogenicity and the initiation of both antibody-mediated immune response and cell-mediated immune responses, VLPs are artificially created nanoparticles made of a subset of viral components that roughly resemble the structure, size, and surface composition of natural viruses [54,163]. VLPs have been created using a variety of expression platforms, comprising mammalian cell lines, bacterial cell lines, insect cell lines, yeast, and plant cells [55]. VLPs are non-infectious due to the absence of core genetic material, suggesting that they are a safer vaccination platform than several other types of vaccine. They are also a safe and relevant model for conducting viral molecular investigations under BSL-2 circumstances without biosafety protection [161]. VLP vaccines offer the benefits of multivalency, inhaled vaccination possibility, self-adjuvant characteristics, scalable manufacture, and readily maintained temperatures throughout the supply chain, in addition to being safe and effective [56]. The FDA has authorized commercially accessible VLP vaccinations against the human papillomavirus (HPV) and hepatitis B [164] and other VLP vaccines, including those for COVID-19 that are presently under development [161].

An ‘S-protein’ expressing DNA vaccine capable of effectively eliciting NAbs and spike-protein-specific T-cell responses has completed phase-II clinical trials [165]. A native-like trimeric spike protein subunit vaccine candidate has been testified that uses the spike protein fused to the C-terminal area of Iα collagen (human type) to construct a disulfide-bonded homotrimer [166]. The S-trimer, together with another AS03 adjuvant or CpG 1018 agonist adjuvant, is also under active phase-I clinical trials. An mRNA vaccine, BNT162b1 from BioNTech/Pfizer, expressing an RBD-trimer stabilized by the fold on a trimerization domain has been observed to elicit high NAbs and TH1 cell-based responses as well as the protein subunit vaccine-ZF2001, which comprises the RBD-dimer, as the target [117]. SARS-CoV-2 S-2P, which contains proline substitution at K986 and V987 residues, is used as the target antigen in the mRNA vaccines produced by Moderna/National Institute of Allergy and Infectious Diseases (NIAID), BioNTech/Pfizer, and a recombinant Ad26 vaccine produced by Janssen [117,150]. The S-2P in the Janssen Ad26-vectored vaccine (Ad26.COV2.S) and the Novavax protein-based vaccine (NVX-CoV2373) employ this tactic because it has been demonstrated that additional mutations at the S1-S2 polybasic cleavage site from RRAR to SRAG or QQAQ make it resistant to a protease that stabilizes the S-protein more in its pre-fusion conformation [151,152], and Ad26 expressing S-2P has been shown to elicit elevated NAb titers [167]. The mRNA vaccines BNT162b2 from BioNTech/Pfizer and mRNA1273 from Modera/NIAID as well as the protein subunit vaccine NVX-CoV2373 have been found to elicit better T-cell responses in addition to high titers [117,153], indicating that the method of stabilizing the spike protein in its pre-fusion state could be a promising area for the production of the SARS-CoV-2 vaccine [154]. Efforts are in place to utilize the specific exoribonuclease (ExoN) present in the genome of coronaviruses to arrest the mutations, since inactivation of this ExoN has demonstrated a twenty-fold increase in the mutation rates [4,5].

Aside from COVAXIN from Bharath Biotech, other inactivated viral vaccine candidates are currently in phase-III clinical trials and one is in the phase I/II stages of trial. Using the “Whole-Virion Inactivated Vero Cell” derived platform technology, Bharat Biotech developed COVAXIN^®^, India’s indigenous COVID-19 vaccine, also in collaboration with the Indian Council of Medical Research (ICMR) and National Institute of Virology (NIV). The two-dose, ready-to-use liquid presentation vaccination regimen administered at 28-day intervals requires no reconstitution or storage below 0 °C temperature. It is also stable between 2 and 8 °C temperature. This vaccine candidate has the unique benefit that, in the same Phase-I research that produced outstanding safety data, vaccination-induced neutralizing antibody titers were seen with two distinct SARS-CoV-2 strains. The Phase-II/III study has also shown adequate safety sequels and better humoral/cell-mediated immune outcomes. The inactivated vaccine candidate has been shown to elicit excellent NAbs titers with no induction of TH1 or TH2 cell-linked cytokines after vaccination [168,169]. Live-attenuated virus vaccines for SARS-CoV-2 have received little research attention [137]. A new adenovirus vectored intra-nasal vaccine, BBV154, that elicits a wide-ranging immune response, reacting effectively with IgG, mucosal IgA, and T cells in the nasal mucosa, is currently under active investigation at Bharath Biotech. The nasal route is the primary portal of entry, and the nasal mucosa is the most vulnerable infective domain for the SARS-CoV-2 virus. The vaccine candidate BBV154 is, furthermore, non-invasive, needle-free, and carries with it the merit of an effortless ease of administration and no needle-associated risk of injuries and infections [170,171].

## 6. Implications of Changing Patterns of Mutations

Undoubtedly, the existing vaccines have been effective in containing the deleterious consequences of COVID-19. Still, the emerging variants of the SARS-CoV-2 have raised concerns regarding the extent of efficacy of these available vaccines to elicit an efficient immune response. This is more germane after the most recent published reports on ‘Vaccine Breakthrough Infections’ with SARS-CoV-2 strains that denote a potential risk of infection with a viral variant virus, even after successful vaccination. Recent studies amongst a cohort who had taken the two doses of BNT162b2 produced by Pfizer–BioNTech or mRNA-1273 prepared by Moderna have reported two women with a vaccine breakthrough infection [172]. These individuals had developed a variant infection despite proven immunogenicity against SARS-CoV-2. Furthermore, the incidence of a breakthrough infection also showed variability among different vaccinated persons who received different mRNA-based vaccines as a preventive measure against COVID-19 infection [173,174,175,176,177,178,179]. A study conducted on 192,123 participants who got two doses of the mRNA-Moderna-1273 vaccine was complemented with an equal number of control individuals, exposed to two doses of the BioNTech-162b2 vaccine, were observed to develop 878 and 1262 breakthrough COVID-19 infections, respectively [174]. Out of the recorded breakthrough infections, seven proceeded to severe COVID-19 conditions but none to other critical diseases, and one to death in the case of BNT162b2. In contrast, in another cohort, only three cases proceeded to acute-care hospitalization, but none led to death or other severe systemic health conditions [174].

The above findings indicate the higher efficacy of the Moderna mRNA-1273 vaccine. They are observed to be associated with a small incidence of Severe Acquired Respiratory Syndrome-CoV-2 breakthrough infection as compared to the Pfizer BioNTech162b2 mRNA vaccine due to their differences in neutralizing antibody titers [180]. A study published by Tang and colleagues on native residents of Qatar indicates the effectiveness of the BNT162b2 vaccine up to 93.4% (95% CI, 85.4–97.0%) against δ variant-induced fatal, critical, or severe disease, which was comparatively lower than the Moderna mRNA-1273 vaccine, i.e., 96.1% (95% CI, 71.6–99.5%) on ≥14 d after the administration of the vaccine’s second dose [178]. Such differences in the efficacy of the abovementioned two nucleoside modified mRNA vaccines could be a product of several factors. There are variations in the formulation and vaccination regime. In contrast to BNT162b2, which is administered at a rate of 30 mg/0.3 mL (100 mg/mL), mRNA-1273 is injected at a dose of 100 mg/0.5 mL (200 mg/mL) 28 days apart. This means that the dose of the Moderna mRNA-1273 vaccine provides three times more copies of the spike protein mRNA compared to BNT162b2, which may enhance immune responses [152,168,169,170]. Further, to enhance in vivo functionality, cellular uptake, and stable delivery systems, lipid nanoparticle (LNP) is rapidly used to coat nucleic acid vaccines. In the Moderna-based mRNA-1273 COVID-19 vaccine, DSPC, SM-102, PEG-DMG, and cholesterol are utilized as LNP, while Pfizer-BioNtech-162b2 is made up of DSPC, ALC-0315, cholesterol, and ALC0159 [171]. The mRNA-LNP vaccination will stop the mRNA from prematurely degrading and will make it easier for antigen-presenting cells (APCs), such as dendritic cells (DCs), to receive it in their cytoplasm [181]. The choice of LNP, however, affects the mRNA stability and durability, which determines the outcome of the immunological response. Heterogeneous variances in immune responses may result from disparities in lipid properties and lipid concentrations [171], despite the reality that both types of vaccine are known to give their recipients a potent immune response against symptomatic COVID-19 infection, compared to acute hospitalizations and death in the unprotected group.

It is interesting to note that research on mRNA-LNP structures suggests that water, ionizable cationic lipids, and mRNA are all found in the core of LNPs. This prompts crucial queries concerning the mRNA’s potential protection against water [182]. For instance, it is not known if or how the LNP’s ionizable cationic lipids interact with the mRNA. To validate the suggested structure and comprehend the effects, further research must be performed. For instance, it has been determined that it is crucial to research the pH within the LNPs in connection to stability [171,182]. The exact type(s) of degradation that mRNA molecules go through in their final formulation should also be thoroughly examined, as should the possibility that strand integrity might be preserved by sequence change [182]. While there are hints that certain folded configurations are more stable, this might also be connected to the analysis and improvement of the secondary and tertiary structure of mRNA [8,171,181,182].

**Figure 3:**
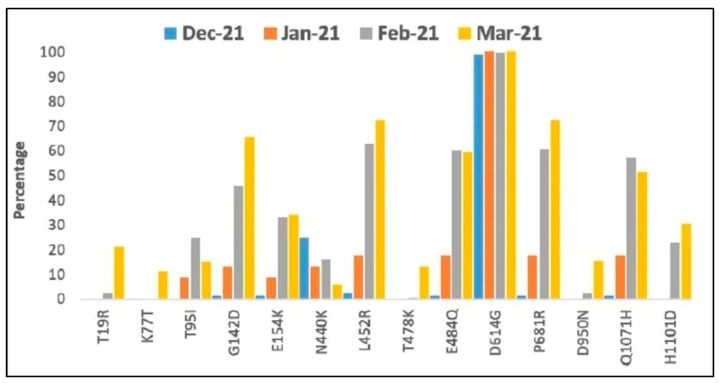
Trend of major mutations in the spike protein from December 2020 to March 2021. [Source: https://www.news-medical.net/news/20210427/Triple-mutation-in-SARS-CoV-2-seen-in-second-wave-of-COVID-19-in-India.aspx, accessed on 15 January 2023].

Since most of the vaccines target the spike protein and the variants have emerged from the same gene S, a big question mark has emerged on the efficacy of these available vaccines to elicit an effectual immune response on these variants, apart from a compulsion to improve the diagnostics and accuracy of the sequencing. The P.1 variant brought about by Spike Protein Substitutions P26S, L18F, T20N, K417T, D138Y, D614G, R190S, E484K, N501Y, H655Y, and T1027I [Figure 3], first noticed in Brazil and Japan, is yet another example of a COVID-19 variant displaying decreased neutralization by post-vaccination and convalescent sera, apart from B.1.1.7, B.1.351, B.1.427, and B.1.429, ruled out by the CDC as variants of concern. The variability in such major mutations has shown an inconsistent trend in the past years; interestingly, the D614G mutation has been reported as an important mutation in the VOCs [Figure 3].

The city of New York, NY, USA, has documented a manifold increase in the cases of variants, especially the B.1.1.7. It is important to consider the factors such as availability of medical care facilities, diagnostic facilities, misdiagnosis, and delay in detection of the disease in asymptomatic patients in determining the vaccine efficacy and difference of fatality rate among different nations [183,184]. The capability of these variants to escape the vaccine-elicited immune response, thereby causing asymptomatic and symptomatic infection spread, is a public health complication of grave concern [172].

However, a very recent (March 2021) study has shown that an inactivated Severe Acquired Respiratory Syndrome-CoV-2 vaccine, BBV152/COVAXIN, can neutralize B.1.1.7 variant considerably [185]. Though the variant has 17 mutations in the genome, 8 of them are located in the spike (S)-RBD that facilitates the binding of the virus to the ACE2 receptor in the host. The study has elucidated that the mutation N501Y, at position 501, with a tyrosine replacement instead of asparagine, upsurges the binding capacity of SARS-CoV-2 to human Angiotensin Converting Enzyme 2, and since many vaccine candidates solely target just one epitope of the original D614G ancestral spike sequence, it is feasible that they are unable to stimulate an immune reaction against the subsequent versions. As a consequence, the study adopted a strategy that involved painstakingly isolating and sequencing the hCoV-19/India/20203522 SARS-CoV-2 (VOC) 202 012/01 from UK returnees to India, which comprised all signature mutations of the UK variant (VOC 202012/01 hallmarks belong to the GR clade of the viral isolates recovered from the UK returnees).

The SARS-CoV-2 strain, i.e., NIV-2020-770, retrieved from tourists arriving in India, has been used for the development of the BBV152 COVAXIN vaccine. The “Vero CCL-81” cells were used for the viral isolation, and the genome sequence was deposited in the GISAID (EPI ISL 420545). The Asp614Gly mutation, which causes glycine to shift from aspartic acid at the 614 amino acid (AA) spike protein position, is present in the BBV152 vaccine candidate strain, which belongs to the G clade and was used for the study’s PRNT50 experiment. Previous studies by the same team have also reported the incapacitated whole-virion SARS-CoV-2 vaccine BBV152, which could elicit a significant neutralizing antibody titre in Phase-I clinical trials against hCoV-19/India/2020770 (homologous) and two heterologous strains from an uncategorized cluster i.e., hCoV-19/India/2020Q111 and hCoV-19/India/2020Q100 that comprise the L3606F mutation. The vaccine has been shown to display significant results for PRNT50 assay (Plaque Reduction Neutralization Test) with 98.6% seroconversion rates for NAbs in Phase-II clinical trial, subsequent to a two-dose immunization plan (0 and 28 days) with a six to eight micrograms antigen with TLR7/TLR8 agonist imidazoquinoline adsorbed on aluminium hydroxide gel.

The NAb titres obtained by PRNT50 of the sera collected 28 days after day 28 of the second dose from 38 recipients of the BBV152 vaccine candidate in Phase-II trial has evidently recognized the efficacy of the BBV152 vaccine against the SARS-CoV-2 UK variant, with (VOC) 202 012/01 hallmarks belonging to GR clade and strain hCoV-19/India/2020770 belonging to G clade. Additional PRNT50 test assay evaluation of 20 representative serum samples from vaccine recipients against the heterologous strains hCoV-19/India/2020Q111 (unclassified cluster) revealed uniform equivalent NAbs titres to the homologous strain hCoV-19/India/2020770 and two heterologous strains, including the distinctive N501Y substitution of the UK variant, hCoV-19/India/20203522 (UK strain), and the hCoV-19/India/2020Q111 as well (for all the samples). These sample sera showed NAbs titre that were equal to the hCoV-19/India/2020770 homologous strain and two heterologous strains, including the hCoV-19/India/20203522 (UK strain) and the hCoV-19/India/2020Q111, both of which had the distinctive N501Y substitution of the UK variety. When compared to the mutant hCoV-19/India/20203522 (UK variation), the median ratio of 50% neutralisation of sera was 0.8, and when compared to hCoV-19/India/2020Q111, it was 0.9 [185,186].

Amid reports of poor neutralization of the UK variant (with E484K substitution) by high NAbs in convalescent plasma, raising a serious question on the global COVID-19 vaccination initiatives, the study has lucidly demonstrated palpable evidence of neutralization of a variant by the vaccinated sera. The most significant aspect of the study is that the vaccinated sera could neutralize the heterologous strains, to, with equal efficacy. Similar studies to evaluate the neutralizing competence of the sera immunized with the mRNA-1273 vaccine have also reported an efficacious neutralizing response against the B.1.1.7 variant [64,186].

## 7. Conclusion and Future Prospectives

The variants of COVID-19 have not only compounded the risk of the infection further but also complicated the diagnostic and therapeutic aspect of the pandemic with reports on ‘Vaccine Breakthrough Infections’. Every vaccine candidate against SARS-CoV-2 is a product of an extraordinary human effort amidst an unprecedented raging pandemic in a short period. Each vaccine candidate carries with it a merit of its own. A conventionally defined ideal candidate vaccine elicits high titers of Nabs and diminishes the production of non-NAbs, thereby reducing the potential of ADE incidence, producing good TH1 cell- responses yet low TH2 cell- responses, reducing enhanced respiratory disease, prompting an enduring immunological memory, and also possesses cross-protection capabilities. Though it is good to conceive and work on a vaccine target cocktail and several other stratagems, several million people are invested in vaccine research across the world, and every vaccine rollout is time-bound, with every rollout going through several stages of cumbersome clinical trials and patient safety protocols. Thus, every vaccine breakthrough infection is quite significant and a considerable challenge to vaccination programs. This is more so in third-world economies.

Thus, the variant challenge mandates more precise and quicker diagnostic tools as well as rapid therapeutic responses to effectively arrest the further spread of infection by the variant. Experience with COVAXIN^®^ and other inactivated vaccine candidates shows us that inactivated whole virus vaccines that could be constructed with little or no complex molecular interventions or alterations in a short period could be an effective manoeuvre to tackle the variants. In fact, these vaccines could be administered as booster shots after the initial vaccination program in regions that have already completed the vaccination for the reference virus. The intranasal vaccine is another promising avenue that confers immunity at the nasal mucosal domain, a vital arena in the infection episode of Severe Acquired Respiratory Syndrome-CoV-2 and additional respiratory viruses with ease of administration of the vaccine.

## Figures and Tables

**Table 1:** Showing the various categories of the variants of SARS-CoV-2 with their clades and origin information.

Categories	WHO Label	Pango Lineage	GISAID Clade	Nextstrain Clade	Area of Documentation	Time of Documentation
	Epsilon	B.1.427/B.1.429	GH/452R.V1	20C/S.452R	United states of America	March-2020
	Zeta	P.2	GR	20B/S.484K	Brazil	April-2020
Variants of Interest (VOIs)	Eta	B.1.525	G/484K.V3	20A/S484K	Not defined	December-2020
	Theta	P.3	GR	20B/S:265C	Philippines	January-2021
	Iota	B.1.526	GH	20C/S:484K	United states of America	November-2020
	Kappa	B.1.617.1	G/452R.V3	21A/S:154K	India	October-2020
	Alpha	B.1.1.7	GRY	20I (V1)	United Kingdom	September-2020
	Beta	B.1.351	GH/501Y.V2	20H (V2)	South Africa	May-2020
Variants of Concern (VOCs)	Gamma	P.1	GR/501Y.V3	20J (V3)	Brazil	November-2020
	Delta	B.1.617.2	G/478K.V1	21A, 21I, 21J	India,	October-2020
	Omicron	B.1.1.529	GR/484A	21K, 21L, 21M, 22A, 22B, 22C, 22D	First lineage reported in South Africa	November-2021
Variants of High Consequence (VOHCs)	None of variant of high consequence has been recorded					

## Data Availability

All data are available in this manuscript.

## Abbreviations

ACE2	angiotensin converting enzyme 2
APCs	antigen-presenting cells
BCR	B cell receptor
BALF	bronchoalveolar lavage fluid
COVID-19	coronavirus disease 19
mAbs	monoclonal antibodies
NAbs	neutralising antibodies
NTD	N-terminal domain
RBD	Receptor binding domain
TH1	T helper 1
TH2	T helper 2
